# Valuing Climate Change Impacts on Human Health: Empirical Evidence from the Literature

**DOI:** 10.3390/ijerph6020759

**Published:** 2009-02-23

**Authors:** Anil Markandya, Aline Chiabai

**Affiliations:** Basque Centre for Climate Change BC3, Gran Via, 35 – 2, 48005 Bilbao, Spain; E-Mail: anil.markandya@bc3research.org

**Keywords:** Climate change, health impacts, adaptation, cost-effectiveness, cost-benefit analysis

## Abstract

There is a broad consensus that climate change will increase the costs arising from diseases such as malaria and diarrhea and, furthermore, that the largest increases will be in developing countries. One of the problems is the lack of studies measuring these costs systematically and in detail. This paper critically reviews a number of studies about the costs of planned adaptation in the health context, and compares current health expenditures with MDGs which are felt to be inadequate when considering climate change impacts. The analysis serves also as a critical investigation of the methodologies used and aims at identifying research weaknesses and gaps.

## 1. Introduction

Climate change is expected to have considerable impacts on human health. The most important include increased vector-, water- and food-borne diseases; injuries and deaths caused by extreme hydro-geological events; heat-related diseases and deaths caused by heat waves (thermal stresses); and negative impacts on malnutrition [1,2]. These negative consequences might be compensated by some beneficial effects, such as fewer deaths from cold weather in temperate regions, but the overall impact is expected to be negative. Evidence suggests that developing countries, including those of sub-Saharan Africa are expected to suffer major health impacts from climate change.

In order to reduce these negative health impacts, various programs or measures, known in the climate literature as adaptation measures, are being put in place or are being planned. Adaptation is defined by IPCC (Intergovernmental Panel on Climate Change) as the “adjustment in natural or human systems in response to actual or expected climatic stimuli or their effects, which moderates harm or exploits beneficial opportunities” [1]. The issue of adaptation to the health impacts of climate change is receiving considerable attention in scientific research and policy debate, particularly after this last report of the IPCC, and is emerging as a policy priority. It is also recognized that adaptation should be more and more integrated into development policies and decision-making processes. This is particularly important for developing countries, where the potential to adapt is constrained by a lack of resources, low health expenditure, weak health care systems and poor health status of the populations. In this context, international assistance for adaptation is foreseen in the least developed countries and small island developing states. Sources of funding include the Global Environment Facility (GEF) under the UNFCCC (e.g. Least Developed Countries Fund and Special Climate Change Fund) and the Adaptation Fund (AF) under the Kyoto Protocol, aiming to support adaptation for food security, disaster prevention, water accessibility and disease control.

For informed decision-making, national governments need therefore to know what financial resources are needed for adaptation, what is the financial gap in funding, what damages could be avoided through adaptation, and what is the cost-effectiveness of the proposed programs. This would allow them to set priorities and to choose the most appropriate combination of measures to reduce the negative health impacts.

This paper critically reviews a number of studies on the costs of health interventions. It analyses the methodological approaches used and identifies critical gaps and research priorities in this area. The goals are three-fold. First, we wish to explore the economic impact of climate change in developing countries in terms of planned adaptation costs, based on the empirical evidence from the existing literature. Quantification of the health costs of adaptation requires an extensive analysis of long-term data including the increase in the number of cases and deaths caused by climate change, as well as economic variables such as unit costs of specific health measures. Such an analysis is more complex in developing countries, due to the lack of consistent long-term datasets. Given these difficulties and the limited number of studies in the context of health adaptation, the analysis is completed by selecting a number of relevant studies coming from the public health literature for climate-related disease control programs. Second, we use the estimated total costs resulting from the literature to perform a simple cost-effectiveness analysis of different health interventions for different diseases, in order to identify the alternatives with the lowest cost to achieve the desired result. Preventive and reactive measures for different health endpoints are compared, the first aiming at avoiding deaths or episodes of illness, while the second are intended to treat the disease when already occurred and minimize the damage. Third, this analysis serves also as a critical investigation of the methodologies used for cost assessment with the purpose of identifying the research gaps and the conditions that need to be satisfied to assure reliable estimates.

Section 2 presents a brief overview of the main health impacts caused by climate change with a specific focus on developing countries. Section 3 presents a classification of adaptation measures, identifies the main cost categories and reports on the methodological approach used for cost assessment. The methodological issues that arise in judging whether a measure is justified from an economic point of view are discussed in Section 4, reporting on two main approaches, cost effectiveness analysis and cost-benefit analysis. Section 5 details the existing studies on the costs of health interventions to reduce climate-related diseases, comparing results among macro-geographical regions. In Section 6 different health interventions are discussed in term of their cost-effectiveness. Finally Section 7 discusses the existing research gaps, the main methodological issues in the cost assessment and how to improve knowledge.

## 2. Health Impacts of Climate Change

Climate change will have a wide range of implications to human health. These include thermal-related morbidity and mortality due to extreme temperatures, effects associated with air pollution, impacts of extreme weather events, malnutrition, water-borne (e.g. diarrhea, cholera, typhoid), food-borne (e.g. *Salmonella*) and vector-borne diseases (e.g. malaria, dengue).

The increase in the frequency and intensity of extreme temperatures will have both direct and indirect effects on health. Direct effects include thermal stresses (cardio-vascular and respiratory diseases, heat exhaustion, heat cramps and dehydration), while indirect effects are related to the impact of heat extremes on urban air pollution and humidity (which can aggravate pre-existing morbidity).

The risk of heat-related morbidity and mortality would increase mainly in the elderly, children, those with pre-existing cardio-vascular and respiratory diseases, and among the urban poor. The greatest impact is expected for mid- to high-latitude cities, especially in poor countries, characterized by poorly adapted buildings and without air conditioning. Lack of datasets and long-term statistics in developing countries makes it difficult to provide quantitative estimates of health effects in these countries. Nevertheless some studies exist which project large impacts in the temperate and tropical regions in Asiaand India [2–4].

The negative impact of heat waves might be partially compensated by a decrease in cold-related deaths during the winter season. According to some studies, the decrease in winter mortality in temperate regions might be greater than the increase in heat-related mortality expected in summer [5,6]. These results are still under debate, as the projected mortality due to cold-related stresses might be overestimated if the effect of influenza is not considered [7]. Further research is needed in this context to understand how the results vary under different climate and socio-economic scenarios. The health benefits are nevertheless expected only in temperate regions, while globally they will be greatly compensated by the increased impact of other diseases, specifically infectious diseases and malnutrition in developing countries.

Extreme temperatures may also increase the exposure to urban air pollution, with the potential to aggravate pre-existing respiratory and cardio-vascular diseases. In the recent years, extreme weather events, such as floods and landslides, storms, cyclones and droughts, have caused considerable damage and loss of life in China, Venezuela, Bangladesh and Mozambique. Direct impacts of extreme weather events include increased incidence of deaths, physical injuries and psychological stresses, while indirect impacts are related to increased risk of exposure to water-borne diseases due to water contamination, and impacts on malnutrition due to loss in agricultural production. Unsafe water and sanitation conditions and decrease water accessibility would further increase the transmission of infectious diseases. It is estimated that by 2050, one billion people in Asia could have limited access to drinking water [8].

Other indirect impacts of changes in weather patterns include variation in the geographical and seasonal distribution of vector-borne diseases (malaria, dengue, leishmaniasis, lyme disease, tick-borne encephalitis), in some cases by expanding transmission to higher altitudes and latitudes. There is, however, considerable uncertainty as to the magnitude of the impacts. Some mathematical models project a net increase in the amount of population exposed to malaria and dengue transmission as a result of climate change [9], while other models suggest only little change in malaria distributions [2].

Finally, higher temperatures can lead to increased exposure to food-borne diseases due to contamination of food, which can cause various gastrointestinal diseases. Notwithstanding the existing scientific uncertainties, most researchers project that in the next decades these health impacts are likely to increase considerably in developing countries, which are more vulnerable to climate change impacts and less capable to adapt to such changes, having poor health care systems, poor health status, and few financial, social and technological resources to adapt. Hence climate change is threatening the capacity of these countries to attain the United Nations Millennium Development Goals by 2015 (UN 2015). It is therefore crucial to understand how climate change is likely to impact development priorities in poor countries, and to respond with appropriate and cost-effective measures for adaptation.

Table 1 reports major direct and indirect effects of climate change on human health, while Table 2 indicates the main health impacts projected for developed and developing countries together with some indication of their adaptive capacity.

## 3. Measuring the Health Cost of Adaptation Policies: Methodological Approach

### 3.1. Health Adaptation Policies and Categorization of Costs

In order to cope with the adverse health effects of climate change, adaptation measures, plans and programs are put in place. Adaptation is defined in terms of “policies, practices, and projects with the effect of moderating damages and/or realizing opportunities associated with climate change” [11]. Adaptation can be classified according to different criteria or aspects [11]. The most important classification is between autonomous (or private) and planned (or public) adaptation. IPCC defines the first as “adaptation that does not constitute a conscious response to climate stimuli but is triggered by ecological changes in natural systems and by market or welfare changes in human systems”, while the second is “adaptation that is the result of a deliberate policy decision based on an awareness that conditions have changed or are about to change and that action is required to return to, maintain, or achieve a desired state” [12].

Another important classification distinguishes between anticipatory (or proactive) or reactive adaptation. The first implies preventive actions which take place before the impact occurs in order to avoid of reduce the risk of disease and death. This type of adaptation involves long-term decision making and reduces long-term impacts, risk and vulnerability caused by climate change. For this reason, anticipatory adaptation is more effective and involves generally planned interventions. On the other side, reactive adaptation provides an immediate response to climate change. The actions take place after the impact with detection and subsequent treatment of the disease. In the public health sector, adaption actions are usually classified into three categories according to the timing: primary, secondary and tertiary [2,13]. Primary prevention regards health interventions put in place *before* the damage occurs in order to avoid it by reducing exposure (e.g. warning systems, water and sanitation programs, distribution of impregnated bed nets, flood protection structures, *etc.*). When the health interventions are implemented *after* the impact has occurred but before the occurrence of the disease, it is termed as secondary prevention. These include measures such as monitoring and surveillance programs allowing an early detection of the disease (e.g. medical tests), or strengthening the public health care. Finally tertiary measures do not attempt to prevent, but to minimize the health impacts already occurred, e.g. through curative treatments.

Other criteria distinguish between short term and long term adaptation, localized and widespread adaptation [11]. Finally, adaptation measures can be grouped into four main categories, according to their function and form: legislative/regulatory, technical, advisory/educational, cultural/behavioral.

Legislative measures consist of laws, guidelines and regulations put in place by the parliament and government to prevent negative impacts of climate change. Technical measures can be anticipatory or reactive, they can be implemented by various government departments depending on the type of measure (e.g. vaccination programs issued by the public health care sector), by the municipality (e.g. green islands in the city centre) or by the industrial sector (e.g. thermal building insulation). Education measures can be implemented by the central government with the cooperation of various departments (e.g. public health). Finally, the behavioral measures are put in place by individuals and they are defined as autonomous adaptation.

Examples of possible health adaptation actions are reported in Table 3. Some of the listed interventions are supported and put in place by the public health sector (like health education activities, or disease surveillance and monitoring). Others, like water treatment and distribution or construction of flood protection structures, are put in place outside the health sector, involving other government departments. Finally, adaptation may include structural and non-structural interventions, where the former, as the name suggests, implies the creation of infrastructures, hardware, house alteration, planning or setting of construction standards (e.g. flood protection structures, water treatment and distribution). Non-structural measures, instead, include all other interventions put in place for treatment or prevention of the disease (e.g. curative treatments, distribution of impregnated bed nets, vaccination, and educational campaigns).

The cost of health adaptation programs includes the cost of building, operating and maintaining health provision service structures, setting up and running emergency response systems, *etc.* We can distinguish different categories of costs, according to the type of adaptation [14]. The first category includes the direct costs related to the implementation of a specific measure (costs of medicines provision and distribution, preventive therapies, *etc.*). The second category involves institutional costs incurred to improve the adaptive capacity of a system. These include actions for strengthening the health care system, infrastructure costs, training activities, communication, monitoring, surveillance and evaluation services, health information systems and population surveys. Finally, the last category includes the transition costs associated with the reallocation of the resources in the society during the adaptation process (e.g. shifts of financial resources to other markets). Transition costs are often excluded from the analysis, due to the difficult estimation process.

From an economic point of view, we should estimate the social costs of adaptation programs. These include external costs incurred by the society (opportunity costs), besides the private costs of production. The studies analyzed in this paper focus on the first two categories of costs mentioned above, namely direct and institutional costs, excluding transition and opportunity costs.

The costs of health adaptation are estimated using market-based data and information in order to perform a cost assessment based on unit values multiplied by the target population living in the vulnerable areas and by the incidence of diseases. Health adaptation costs include treatment costs of additional cases (reactive adaptation) and costs for preventive measures to reduce the incidence of disease (proactive adaptation). The cost of health adaptation will depend on the health outcome, the intervention type (e.g. treatment or prevention), the expected reduction in the incidence of mortality and morbidity and finally on the geographical region where the impact is expected.

## 4. Use of Evaluation Tools for Policy-Making in Health Contexts

As important as estimating the costs of adaptation programs for health protection, is to have some way of judging whether these costs are reasonable and whether the program is justified. For this purpose economists use two tools: Cost-Benefit Analysis (CBA) and Cost-Effectiveness Analysis (CEA). The main difference between CBA and CEA is that in CBA both the costs and benefits of a proposed program are estimated in monetary terms, and then compared with the costs and benefits of alternative programs, in order to identify the program with the highest net benefits. By contrast, CEA estimates only the costs of attaining certain health goals, which are measured in physical units (number of lives saved, number of cases of illness avoided, and quality adjusted life years - QALY).

### 4.1. Cost Effectiveness Analysis

Cost-effectiveness analysis (CEA) is a technique used to identify the least cost intervention to reach a desired result. It enables us to define priorities among different alternative interventions by identifying the least cost option able to reach the underlying objectives. CEA is sometimes used as a second-best option when a full-blown CBA would be desirable, but many benefits cannot easily be monetized [15].

The cost-effectiveness ratio of a program is computed by dividing the annualized costs of the program by the physical benefits, measured in terms of lives saved (or life years saved), and cases of illness avoided. Results are expressed as unit costs, in terms of costs to be supported to save one life (or one year life) and to avoid one episode of illness. If all else is the same, the program deemed more cost-effective would be the one with the lowest cost per life saved or avoided case. For example a government might decide that any project where the cost per life year saved is in excess of €1,000 cannot be funded. It could arrive at this conclusion, given information on the range of possible interventions, the cost per life year associated with them and the budgetary resources available. Each project then would have to report a cost per life year saved and a necessary condition for adopting it would be that this cost is less than €1,000. This approach is attractive because: (a) it is generally feasible (although not always easy) to calculate the cost per life year saved or case avoided from health related interventions and (b) the criteria are simple and transparent. Traditionally, CEA has been used widely in medical decision-making and in public health although in practice the selection of health interventions does not rely exclusively on this criterion [16].

Estimation of the costs of a program within a CEA is similar to that in a CBA. The advantage of the first is that the benefits are computed in physical terms and not in monetary terms as in a CBA, which results in a less complex calculation. Analysts engaging in a CEA, however, still have to deal with complex issues such as the choice of the discount rate when the costs of the program are incurred over several periods. Further problems arise when a program impacts on more than one indicator (e.g. it results in savings in life years as well as a reduction in the experience of unpleasant symptoms). In such cases other criteria have to be used or one has to be chosen at the expense of the other This is particularly relevant as a program could reduce both mortality and morbidity impacts, and in this case the use of one indicator (cost per case avoided) instead of the other (cost per life saved) could have misleading results if the two measures differ widely. In these cases another physical indicator known as the Quality Adjusted Life Year (QALY) is routinely used in medical decision-making and public health. This measure is based on the individual preferences over quality of life and longevity, which are assumed to depend on the health consequences (it is assumed that a year of healthy life expectancy is worth 1, while a year of unhealthy file expectancy is worth less than 1, being this value lower the worse the quality of life). This indicator has the advantage of incorporating both mortality and morbidity effects, instead of favoring one of the two. The main problem with this approach is that older individuals would have fewer QALYs than younger individuals, which would lead to higher cost per case avoided or per life saved for the elderly. Furthermore this approach does not account for individual risk perception. For these reasons, in our computations we use the indicator cost per case and/or death avoided.

In the context of climate change CEA is a useful tool to provide a measure of the costs of a program in terms that are comparable across programs. Studies on the costs of adaptation programs are indeed difficult to compare in a way that provides some guidance on whether they are justifiable or not. Although imperfect, measures such as the cost per case avoided or cost per life year saved are at least feasible and the numbers obtained useful for policy purposes.

### 4.2. Cost Benefit Analysis

This technique has been used to analyze many public policies, including transportation, urban regeneration, agriculture, public health, education, and the environment. It is also used for valuing for adaptation to climate change impacts. By monetizing the benefits of a program, CBA makes it possible to evaluate many different types of government programs and to compare them with the costs of the programs. When different programs are examined, economists suggest choosing the one with the largest net benefits (equal to total benefits minus total costs of the policy). Note that, unlike CEA, cost-benefit analysis also allows one to determine the socially optimal size of the program, i.e., the one that maximizes net benefits.

In order to determine the net effect of a proposed program, we must first identify who will gain (beneficiaries) and who will lose from its implementation, and then estimate the gains or losses for each of the partners. In spite of that equity considerations are not sufficiently reflected in CBAs (the argument that projects or policies with the best benefit-cost-ratio are socially desirable rests on the assumptions that the gainers can – in principle – compensate the losers of a project/policy and still be better off. Since such compensation is not generally paid, most projects or policies have distributional implications that need to be taken into account). CBA is complicated further by the fact that, while it is quite easy to identify the costs, it is more difficult to estimate the benefits, as these are generally non-market goods, for which no market prices exist.

Another problem with CBA is that it must compare costs and benefits that are experienced at different times in a single measure. The choice of the rate at which future costs and benefits should be discounted is particularly relevant for climate projects because costs of adaptation measures will be incurred in the near future while the benefits will be spread over a much longer time period. The debate on discounting for climate change is not resolved but many economists argue that very long term impacts of the kind associated with climate change require very low discount rates – of the order of one percent or less. In practice normal public sector projects involve discount rates of around 5–6 percent [18].

One other feature of the use of CBA to evaluate health adaptation programs is worth mentioning. This is the problem of valuing benefits that take the form of reductions in mortality – implying savings in life years, which in turn requires a value to be attached to a life year or a life saved. In the past this has produced serious debate and disagreement. Methods have been developed for making such estimates, but the studies are mainly from developed countries. In the context of climate change the use of different values for a life year according to the country in which the person lives has raised serious problems (this issue created a political crisis at the time of the 2^nd^ IPCC Report when a recommendation to take a lower value of life in developing countries compared to industrialized was strongly objected by representatives from developing countries). Studies in the climate change field have wrestled with this problem and resolved it with broadly two uneasy compromises. Some have taken different values for different countries, more or less in proportion to real per capita GDP [17]. Others have applied the same value to all lives saved, irrespective of the country in which the person is resident [18]. Neither is really satisfactory and a number of scientists working in this field feel that this issue is itself enough to avoid using CBA in the context of climate change (however some targeted use of CBA may be appropriate and useful when for example an intervention has multiple benefits and the monetary estimation of the non-health benefits is strong). Given the difficulties in estimating health benefits in monetary terms, we have used the cost per case avoided and cost per life saved to compare effectiveness among different health programs.

## 5. Assessing the Costs of Health Interventions in Developing Countries: Empirical Evidence from the Literature

The analysis reviews a number of valuable studies about the costs of health interventions (planned actions) to reduce the disease incidence and related mortality, with a specific focus on developing countries, expected to face most of the health impacts of climate change. The studies selected focus on similar targets in terms of health impacts reduction, which allows comparison among interventions types and countries. The health outcomes considered in the analysis are vector-borne and water-borne diseases, malnutrition, pneumonia and newborn causes of death in children. It must be recognized that quantification of costs is much more difficult in developing countries, due to the lack of data and long-term analysis, high variation of data among countries, inefficiencies in disease detection and reporting. Nevertheless we have selected some relevant studies in order to analyze the methodology and to draw some quantitative estimates of the cost of health adaptation.

There are two main groups of studies considered in this analysis. The first relate to health care costs associated with the additional health impacts arising as a result of climate change. These costs include disease treatment measures (reactive adaptation) as well as preventive measures to reduce the incidence of climate related diseases (anticipatory adaptation). Despite the important role of adaptation in reducing the health costs of climate change (known as the ‘costs of inaction’), there is very little information available from the literature on the costs of health adaptation in different climate change scenarios. For this reason we have reported on a second group of studies focusing on the costs of health interventions programmed to achieve a reduction in the burden of climate-related diseases, in accordance with internationally agreed objectives and targets, like the MDGs. These costs are estimated outside the climate change context, but they focus on climate-related diseases (e.g. malaria, diarrhea, pneumonia). As the types of measures used for disease treatment and prevention are, in principle, the same in both contexts, the second group of studies can be reasonably used to provide some indications of how much adaptation measures would cost.

*The above mentioned studies focus on planned interventions only as they aim at identifying the financial resources needed by the government to address the increased impact of climate-sensitive diseases. Autonomous adaptation, which includes costs supported by households, is not taken into account. The figures discussed in this paper represent therefore a lower-bound estimate of the total costs of health adaptation supported by the society as a whole. Household costs include direct expenditures for health for both prevention and treatment (medical care costs, medicines) and indirect costs such as loss of earnings due to absence from work, inability to perform usual activities, need to care for children, time spent travelling and, in case of premature death, the discounted future lifetime earnings or the value of a statistical life. These studies are not included in the present review as the methodological approaches used for estimating these impacts rely on different theoretical backgrounds, raising specific research issues. Methods used in this context include cost of illness, compensating wage studies and contingent valuation*.

### 5.1. Costs of Health Adaptation Due to Climate Change Impacts

The methodological approach for estimating health adaptation costs requires to estimate the disease incidence due to climate change, to project future population under different scenarios, and to estimate the number of people at risk in the future (multiplying future population with the incidence ratio). The costs of adaptation are assessed by multiplying the number of people at risk in the future with the cost per capita of health interventions.

Perhaps the most important study in this category is that of Ebi [19] who estimated the costs of specific interventions for treatment of additional cases of malaria, diarrhoea and malnutrition expected to occur between year 2000 and 2030, due to climate change. These were estimated under three exposure scenarios related to climate change: unmitigated emission trends, stabilization at 750 ppm CO_2_ equivalent by 2210 and stabilization at 550 ppm CO_2_ equivalent by 2170. Estimates are provided separately for Africa, North and South America, South-East Asia, Europe, Eastern Mediterranean countries and Western Pacific countries. The estimated figures do not include the full range of costs [20]; for example infrastructure, equipment and health care personnel costs, training costs and maintenance costs are excluded from the analysis. For diarrhoea, the costs included in the analysis are those related to immunization programs and improvement in water supply and sanitation. As regards malaria, the estimated measures include provision of impregnated bed nets, indoor spraying, and preventive treatment in pregnancy, besides the specific required therapies. Finally for malnutrition, the approach used is very conservative, as only the costs of nutritional programs and monitoring have been included. For all their gaps, however, these estimates are probably closest available to the desired costs of adaptation.

Total costs under various categories for the year 2030 are summarized in Table 4. The figures show malaria as the most significant, representing just under over half the total costs, followed closely by diarrhea, which accounts for just under half. Malnutrition related illnesses are only a minor component, making up just one percent of the total, but we should consider that this is a lower bound estimate and that most of the impacts have not been calculated.

For the sake of comparison among different countries, additional annual costs have been calculated for major geographical macro-regions in developing and developed countries. Detailed estimates for these macro-regions are reported in Table 5 below only for diarrhoea and malaria, which account for the majority of total costs. As expected, developed countries account only for a minor share of total costs (around 3–4% for diarrhoeal diseases, and 0.008% for malaria), while developing countries will bear almost all the projected additional costs.

As regards diarrhoeal diseases, South East Asia and Africa are expected to face the highest costs in all scenarios, accounting for respectively 42% and 27% of total annual costs for health interventions in developing world, followed by Western Pacific with 16%. As for malaria, the majority of the costs are expected in Africa (82%), followed Eastern Mediterranean countries (14%).

In terms of gains from reducing GHGs in developing countries, Table 4 shows that we can achieve a 25% to 35% reduction in costs for treating diarrhoeal diseases, and 37% to 49% reduction in costs for malaria by reducing GHGs enough to stabilize emissions at 750 ppm and 550 ppm respectively. Whether or not this is justified will depend of course on the costs of making the reductions in emissions as well as the other benefits of those reductions.

The second study in this context is from Van Rensburg and Blignaut [21] who have estimated the additional health care costs in year 2025 due to an increase in the incidence of malaria as a result of climate change in Southern Africa. The analysis focuses on prevention and treatment costs of malaria. The incidence ratio has been calculated for different scenarios (from no risk to low and medium risk, from low and medium risk to high risk, and increased risk for high risk areas). Projections of future population are based on low population growth in the region because of the expected impact of HIV/AIDS. For cost assessment, results from Mills [22] are used and adjusted to 2000 prices using purchasing power parity. Total annual costs for prevention and treatment of malaria in South Africa are estimated around US$ 3,800 million in 2025 (2000 US$) (Table 8) (the upper-bound cost estimates are reported which include all the measures required for disease treatment and prevention), representing 3% of GDP per capita. Costs have been estimated also for Botswana and Namibia, where the figures are lower because of the smaller population (US$ 125 million in Botswana; US$ 177 million in Namibia). Namibia is expected nevertheless to face the highest cost in terms of percentage of GDP/capita estimated around 4.5%. These figures are more conservative but still comparable with the results of Ebi [19] for malaria, where the valuation focuses on a somewhat larger timeframe and on all the African regions.

The above mentioned figures can be compared with the total expected costs of malaria if no intervention was implemented [23], which was estimated in 1999 around US$ 90–270 billion without considering climate change impact (estimates are based on the observed number of cases without interventions; Murray and Lopez [24] have estimated around 36 million lost DALY for malaria in 1999 in sub-Saharan Africa; two estimates of DALY have been considered: the first equal to the per capita income, and the second valued at three times the per capita income). This cost would be much higher in the presence of climate change, which is expected to increase the relative risks of malaria, and clearly it would be well beyond the budgetary capacity of governments.

### 5.2. Costs of Climate-Related Disease Control Programs in the Public Health Context

In this section we analyse and discuss the results of some studies estimating the costs of health interventions outside the climate change context but focusing on climate-related diseases and therefore relevant to the climate change health discussion. The diseases looked at are malaria, diarrhoea, malnutrition, pneumonia and other newborn diseases. The studies included in the analysis report estimates for macro-geographical regions and for a combined set of intervention programs.

The first study analysed is Kiszewski *et al.* [20] who estimated the overall financial resources needed to implement preventive and curative measures to reduce malaria by at least 50% by 2010 and 75% by 2015 in the most affected malaria-endemic countries (in Africa, Asia and Middle East, and South America), according to the recommendations stated by the World Health Assembly in 2005. The study reports the additional financial needs to cope with these targets, together with a comparison with the national governmental resources currently available for malaria control. The estimated resources have been calculated by gathering data from a set of widely recommended interventions. These include (i) prices of selected products for prevention, diagnosis and treatment of malaria, including the costs of distribution, (ii) costs of vector control and personal protection (insecticidal nets, spraying), (iii) control of epidemics, (iv) costs of preventive treatment in pregnancy; (v) management of severe cases and costs of rapid diagnostic tests, (vi) costs of strengthening the health care sector (train personnel and equipment, transport facilities and logistics improvement), (vii) communication and information costs, (viii) cost of prevention, including monitoring, surveillance and evaluation services. Cost estimates in this study include therefore the full range of costs.

The study integrates cost valuation methodologies with epidemiological estimates about the proportion of people exposed to malaria (using climatic modelling and clinical evidence of incidence) and projections of population growth rates (unit costs have been calculated in each country for the baseline year. For preventive measures, unit costs are multiplied by the total population living in areas at risk. For reactive measures, unit costs are multiplied by the incidence of disease in those areas. Future changes in demand and production have not been considered in this analysis, nor have the associated effects on future prices).

Results show that the total financial resources needed from 2006 to 2015 to implement the recommended measures (in Africa, Asia, Middle East and South America) amount to US$ 38 to US$ 46 billion (on average US$ 3.8 to 4.6 billion per year). Among the different cost categories, the vector control costs are estimated to be the highest. The average annual costs (2006–2015) are expected to be around US$ 1.7–2.2 billion for Africa, around US$ 1.9–4.6 billion for South East Asia, Western Pacific and Eastern Mediterranean, and finally US$ 212–235 million for Central and South America (Table 6).

These figures are similar to the costs for health adaptation estimated by Ebi [19] (although the two do not measure exactly the same things). In Table 5 addressing the costs of additional cases of malaria in 2030 is estimated at between US$ 3 to 8.8 billion in the absence of any program to reduce GHGs (Table 5, last column). Thus the bottom end of the figures from Ebi are similar to the bottom end of the costs of reducing malaria by 75 percent in 2015, but the Ebi estimates are more nearly double the Kiszewski [20] figures at the top end of the range. Indeed, a telling feature of the costs of adaptation is their wide range.

If we compare total costs of these interventions with the national resources available for malaria control we find that only 4.6 percent of the estimated annual financial resources required can be covered by national resources in the African countries, and only 9.2 percent in the countries of Asia, Oceania and Americas, showing a financial gap between domestic funding and funding needs on annual basis of 90–95 percent. This suggests that governments are unable to fund programs at a level high enough to meet the malaria targets or to treat the additional cases resulting from climate change.

The results of Kiszewski *et al.* [20] can also be compared with the estimates for malaria treatment and prevention provided by Epstein and Mills [23]. According to this study, the overall additional health care expenditure required in Sub-Saharan Africa to achieve 40 percent population coverage for preventive measures and 50 percent coverage for treatment measures (according to the MDGs 2007) range from US$ 0.65 to 1.4 billion per year for prevention and from US$ 0.6 billion to 1.1 billion per year for treatment. The annual costs rise to US$ 1.4–3.2 billion for prevention, and US$ 1.4–2.5 billion for treatment, if we want to achieve the MDGs targets for 2015 of 70% prevention and treatment coverage. These figures are higher than those estimated by Kiszewski [20] for Africa (who estimate a range from US$ 1.7 to 2.2 billion for both prevention and treatment of malaria to ensure 80% coverage). They are, however, of a similar magnitude to the estimates provided by Ebi [19] under the unmitigated scenario for Africa regions (US$ 2.6–7.2 billion).

Another relevant study for malaria is that of Morel *et al*. [25] who estimated the costs of selected malaria control programs in the context of the Millennium Development Goals. Analysis is focused on two sub-Saharan African regions, particularly vulnerable to malaria: Southern and Eastern Africa (with high child mortality and very high adult mortality for all causes) and Western Africa (with high child and high adult mortality).

Various prevention and treatment measures and combinations of them on a 10 year timeframe are evaluated under different assumption of population coverage. Preventive interventions include vector control programs like insecticide treatment of bed nets, indoor residual spraying and intermittent treatment of pregnant women (aiming at reducing neonatal mortality). Treatment measures include distribution of several drugs, and combination treatments, while hospital admissions are not considered. Interventions have been evaluated at 50%, 80% and 95% coverage. Population at risk is calculated taking into account the proportion of person living in malaria endemic areas. Results show that, for 95% population coverage, annual costs are equal to US$ 468 million for Western Africa and US$ 442 million for Southern and Eastern Africa (based on year 2000 estimation), for a combination of measures including indoor residual spraying, impregnated bed nets, case management based combination therapy and intermittent presumptive treatment in pregnancy. These figures are 45 to 70 percent lower than those provided by Epstein and Mills [23] and Kiszewski *et al.* [20], as some relevant costs are not included in the analysis, like treatment of severe and complicated malaria, training for staff and community, communication costs, monitoring, evaluation and operational research and finally all the fixed costs related to strengthening the existing infrastructures.

A more extensive study estimating health care programs costs is Stenberg *et al.* [26] who estimated the additional resources to implement a set of measures to reduce child mortality and morbidity by two-thirds by 2015 in 75 countries in the developing world, according to the Fourth Millennium Development Goal. The selected countries show high mortality among children, accounting for 94 percent of total number of deaths registered worldwide among children less than five years old. The causes under analysis were malnutrition, diarrhea, malaria, pneumonia and newborn causes of death in children. Estimates include the full range of costs including, among others, costs for immunization, malaria treatment and prevention, insecticide treated bed nets, diarrhea and dysentery management, nutrition intervention, case management of neonatal infections, and finally program costs for support activities (to strengthen the health systems capacity to provide appropriate interventions). Findings suggest total additional resources of US$ 52.4 billion in the period 2006–2015, resulting in additional annual costs of US$ 2.2 billion estimated in 2006, and US$ 7.8 billion in 2015 (estimates are based on the expected number of births by country and year by 2015; the epidemiological model assumes constant risks up to 2015. We should note that the risk of mortality and morbidity are expected to rise as a result of climate change, and this is a main difference with the study of Ebi [19]). This is equivalent to an increase in *per-capita* health expenditure of US$ 0.47 in 2006 and of US$ 1.46 in 2015. The average incremental cost per child less than five years old who is treated is estimated to be US$ 12.31 in 2015. The suggested interventions would require an increase in total health expenditure in the 75 countries of around 8 percent on average and in government health expenditure of 26 percent over 2002 levels. Low and middle-income countries with weak health care systems would experience substantial difficulties to collect sufficient domestic funding to cope with MDG goals.

Hutton and Haller [27] estimated the costs of water and sanitation improvement programs in developed and developing countries. They used five different intervention scenarios to be implemented from year 2000 to year 2015. The health benefits are related to a decrease in water-borne diseases, mainly infectious diarrhea (cholera, salmonellosis, shigellosis, and other intestinal infections). Costs included in the analysis regard increased access of water supply and sanitation for people without access, involving investments and maintenance costs. Investment costs include planning and supervision, hardware, construction and house revision, protection of water sources and educational campaigns. Maintenance costs include operating materials, maintenance of the hardware, regulation and control of water supply, water treatment and distribution. These costs are essentially related to structural interventions outside the public health (known also as environmental health interventions), while the other studies mentioned above focus instead on non-structural public health measures (like curative treatments and prevention based on distribution of medicines and other measures like impregnated bed nets).

Total annual costs of these interventions (in developing and developed countries) range from US$ 1,782 million in the first scenario to a huge US$ 136,514 million in the fifth scenario. The first scenario involves improvement in water accessibility only, while the second scenario involves both water and sanitation accessibility improvement, requiring therefore much larger financial resources (equal to US$ 11,303 million). Investments on sanitation are indeed considerably more expensive. In scenario three it is expected that the entire population has access to water and sanitation services, which doubles the costs of implementation (equal to US$ 22,612 million). Scenario four requires only a low increase in financial resources as it includes the availability of disinfected water at the point of use (US$ 24,648 million).

The high costs for the fifth scenario (US$ 136,414 million) is explained by the substantial investments and corresponding maintenance costs necessary to guarantee access for all, in order to regulated piped water supply and sewage connection in the houses. These figures are much higher than the corresponding annual costs for non-structural interventions for disease control and prevention in public health but the water and sanitation programs do attain a number of other objectives as well. In terms of health they attain a 60–70 percent reduction in the incidence of disease.

Table 7 reports the annual costs for the five intervention scenarios by geographical macro-regions. As expected, the large majority of the costs occur in developing countries, which account for around 95 percent of total costs. Except for the first scenario, the highest share of costs (around 35 percent of the total) is expected in South Eastern Asia regions (Indonesia, Sri Lanka, Thailand, Bangladesh, India, Maldives, Nepal, Bhutan, *etc.*), followed by Western Pacific regions (China, Mongolia, Malaysia, Philippines, Korea, Viet Nam, and Cambodia) with 32 percent and Africa regions with 18 percent of total costs. The number of cases avoided by water and sanitation programs range from 3 percent decrease in the first scenario to 70% decrease in the last scenario.

The last study analyzed in this group is the one of Meddings *et al.* [28] in the context of environmental health interventions. The study estimates costs of a program for latrine construction and renovation in Kabul (Afganistan), implemented by the International Committee of the Red Cross (ICRC) in 2006. The program allows to reduce diarrhoreal diseases. Annual costs have been estimated from US$ 503,948 to 979,301 (1999 US$). This study is not fully comparable with the above mentioned studies as it focuses on interventions to be implemented just in one city. It has nevertheless been included in the review as it provides interesting insights for the cost-effectiveness analysis reported in the next section. The above mentioned studies and cost assessment are synthesized in Table 8.

## 6. Cost Effectiveness of Health Protection Programs

In this section we use the cost estimates from the above-mentioned studies to compute a simple index of cost-effectiveness for different intervention programs and health endpoints. We use two indicators, the cost per death or per case avoided, depending on the available data and the intervention targets and objectives. Only health benefits are incorporated in the index, while potential non-health benefits are excluded from the analysis.

Results are reported in the fourth column of Table 8. The following conclusions can be drawn from these figures. The cost per case avoided of malaria in Kiszweski *et al.* [20] has been computed only for South America, for which we found available data. This cost is around US$ 260–300. It is a matter of judgment whether or not this is justified. One can expect a similar cost for treating cases resulting from climate change.

The cost per case avoided in water and sanitation programs has been based on the data of Hutton and Haler [27]. More details of the costs avoided in that study are provided in Table 9, which relate to interventions in improved water supply and sanitation. The range of US$ 12 to US$ 37 is much lower than that of Kiszewski *et al.* [20], suggesting that programs based on improving water and sanitation (structural interventions outside the public health), even if requiring very high initial investments, are more cost effective than those involving curative treatments and prevention based on non-structural interventions in public health (water and sanitation programs, being structural interventions affecting different sectors, would provide also considerable non-health benefits in terms of time savings for water collection, more efficient water resources management, property value rise, increased leisure activities, benefits to agriculture and industry for improved water supply, and also some development benefits like income-generating technologies for water and sanitation access; their cost-effectiveness would therefore be probably even higher if all the benefits are taken into account). This is explained by the higher number of cases of illness avoided in the former compared to the second.

Finally, we include the cost per death avoided estimated by Meddings *et al.* [28] which is much higher than the cost per case avoided in Hutton and Haler [27]. This can be explained by a number of reasons. First, the two measures are not fully comparable as they are based on different units (number of deaths and number of cases), which can explain part of the difference. Second, only reductions in child deaths are considered in Meddings *et al.* [28], while the other age groups are not counted in the cost-effectiveness ratio. Finally, only reductions of deaths over one year are calculated, while the benefits of the program would last for a number of years. This study has been included in the discussion as it suggests how it is difficult to compare cost-effectiveness among health programs and that the results have to be interpreted with caution. For comparing costs and cost-effectiveness among regions and interventions, it is essential that the studies are fully comparable in terms of spatial analysis (aggregation of geographical regions), temporal analysis and intervention types.

The analysis is completed with a critical review of the existing studies about the cost-effectiveness of single health interventions in developing countries (Table 10) (see also [29,30]). The health outcomes under analysis are diarrhoeal diseases and malaria. For diarrhea, the interventions considered are immunization programs, vitamin A supplementation and breastfeeding promotion. For malaria, both prevention and treatment measures are discussed. Prevention includes vaccination, bed nets impregnation, vitamin A supplementation and prevention in pregnancy. Treatment includes early diagnosis and prompt intervention with drugs.

Results vary according to the intervention type and the country of origin. Cost effectiveness for immunization programs of diarrhea varies from US$ 226 to 887 (US$ 1999) per death avoided, while breastfeeding promotion ranges from US$ 115 to 919 per death avoided. These results confirm that non structural health interventions are less cost-effective compared to structural environmental health interventions like water and sanitation programs [27].

As regards prevention of malaria, cost per death avoided is around US$ 305 (US$ 1999) for vaccination, it ranges from US$ 227 to 858 for bed net impregnation measures, from US$ 73 to 414 for vitamin A supplementation and from US$ 81 to 950 for prevention in pregnancy. Treatment measures of malaria show a cost per death avoided of US$ 271–1,355 for early diagnosis and prompt treatment.

These results are comparable with the cost per death avoided we estimated using data from Kiszewsky [20] and Stenberg [26]. It must be said, however, that these latter evaluated a combination of different measures, while the above mentioned studies focus on single specific interventions, and the resulting costs per death avoided cannot be simply summed up to get an overall cost effectiveness ratio. A set of combined measures is in fact more cost effective than a single measure as it provides a higher reduction in the number of deaths and cases of disease. Of course one should choose the most effective measures first but it is hard to argue that a life is not worth at least that much anywhere in the world.

## 7. Conclusions and Recommendations

In this paper we have looked at the costs of adapting to climate change from a health perspective and we have computed a simple cost-effectiveness index for alternative programs based on the estimated total costs resulting from the literature. The analysis is completed with a critical review of the existing studies about the cost-effectiveness of different health interventions.

There is a broad consensus that climate change will increase the costs arising from diseases such as malaria and diarrhea and, furthermore, that the increases will be largest in the developing world. Estimates of the costs of adaptation measure the additional cases and the costs of treating those additional cases. They may also include some estimates of more preventive actions. One of the problems in this area is a lack of any studies that measure these costs systematically and report the data in detail. The detailed study of Ebi [19] concludes that additional annual costs will be around US$ 3–8 billion for malaria and US$ 3–9 billion for diarrhea worldwide. These figures are for 2030 under the assumption of no mitigation. Under the most optimistic stabilization scenario the costs could come down by as much as 40–50 percent. In the case of Africa, the additional costs in 2030 are estimated at US$ 2.5–7 billion for malaria and US$ 1–2 billion for diarrhea. The second study analyzed in the climate change context shows that additional annual costs would be around 3,800 million US$ in 2025 in South Africa alone.

Although these estimates refer to 2025 and 2030, which may seem a long time away, the additional cases are already evident and climate change is increasing the relative risk of these and other diseases right now. The implications are quite serious in terms of the goals of reducing deaths from malaria and other causes of infant and child mortality (Goals 4, 5 and 6). Estimates of expenditures made in respect of attaining these goals indicate expenditures for malaria alone of 2 billion US$ per annum in Africa (based Kiszewski *et al.* [20]; others indicate somewhat higher figures). If the relative risk is raised as a result of climate change this sum will not be sufficient. And in any case the amounts currently available to address these problems are only around 5 percent of the required budget. So not only are the MDGs in danger of not being met on grounds of insufficient funds, they are also in danger of not being met and of progress being reversed as a result of climate change.

The case for making these expenditures is strong, on economic as well as on moral grounds. One of the studies cited estimates the costs to people who contract malaria and who are not treated at US$ 90–270 billion in sub-Saharan Africa in 1999 [23]. This is almost two orders of magnitude greater than the annual costs of health programs for treatment and prevention (Epstein and Mills [23] estimate that if action is taken to treat cases and introduce other health interventions the avoided costs would range from US$ 87 to 267 billion. The costs without interventions are thus much higher and the expenditures on intervention amply justified. Furthermore if we consider that the rise in global temperatures is expected to increase the relative risks of malaria incidence, the impacts will be all the greater). Furthermore we find that the cost per death avoided through disease control programs focusing on combined health interventions is of the order of US$ 300–600. On moral grounds most of us would find it unacceptable to believe that a life is not worth that much in even the poorest country.

The paper provides finally a critical analysis of the reported studies coming from the public health literature showing a number of relevant points, which should be considered in future research. The magnitude of the costs of health interventions for reducing climate-sensitive diseases (not related to climate change) is comparable to the costs of health adaptation under climate change but the latter have a much wider range. Hence more work is needed to narrow the estimates of the costs of adaptation.

The studies make it difficult to identify which interventions are the most cost effective in targeting diseases related to climate change. A more consistent approach to further work in reporting cost effectiveness indicators would help us make more informed judgments. One problem is the use of different metrics (cost per case or per death avoided). Another is how to consider programs with multiple benefits. As regards malaria, for example, the measures relate to disease prevention and treatment and they provide mainly health benefits. For diarrhoea, instead, structural intervention can be implemented which provide also considerable non-health benefits. These should be also taken into account when computing the cost-effectiveness ratio.

In spite of this we note that the costs per life saved in the case of diarrhoea are considerably lower than those in the case of malaria. That would suggest at least starting out targeting diarrhoea cases. Furthermore, within that group the costs of improved water and sanitation, while requiring huge investments to meet the MDGs, result in costs per case avoided that are fairly low. Given that these same interventions have multiple benefits, not all of which have been accounted for would suggests that non-health structural interventions may be more cost effective but further research is needed to confirm this.

## Figures and Tables

**Table 1.** Health Impacts of Climate Change: Classification.

	Health impacts	
Climate impacts	Direct	Indirect
Temperature extremes (heat or cold waves).	Heat- and cold- related stresses	- Respiratory and cardio-vascular diseases due to the combined effect of exposure to high temperature and air pollutants
Extreme weather events		
Floods, landslides, storms, cyclones	Deaths and injuries	- Water-borne diseases caused by water contamination and poor sanitation conditions - Psychological morbidity
Droughts	–	- Malnutrition and under-nutrition, due to loss of agricultural production - Water-borne diseases caused by decreased water access and malnutrition - Vector-borne diseases due to changes in vector transmission and stagnation/contamination of small rivers and drainage canals - Respiratory diseases due to increased air-borne particulate matter and increased vulnerability caused by malnutrition and other diseases
Increased temperature	–	- Vector-borne diseases due to higher risk of transmission and changes in the geographical and seasonal distribution - Food-borne diseases due to food contamination

**Table 2.** Health Impacts of Climate Change in Developed and Developing Countries.

Region	Health Impacts	Adaptive Capacity
Africa	- Changing in spatial and temporal distribution of malaria, dengue, diarrhea, cholera, meningitis, *etc.* - Increased deaths and injuries due to extreme weather events in new areas -Malnutrition	Low adaptive capacity due to lack of financial and technological resources, low GDP per capita, poverty, limited infrastructure, weak primary health care, high infant mortality, low education levels, limited access to capital, armed conflicts.
Asia	-Thermal stresses due to heat waves in East Asia -Air pollution related diseases -Transmission of malaria to new areas -Increased morbidity and mortality due to diarrhea in South and Southeast Asia and cholera in South Asia -Increased deaths and injuries due to flooding and extreme events in East Asia, Southeast and South Asia -Malnutrition	Adaptive capacity varies among countries and is often constrained due to poor financial and technological resources, income inequalities and weak health care system.
Latin America	-Thermal stresses due to heat waves in big cities -Transmission of vector-borne diseases to new areas, including malaria -Increased deaths and injuries due to tropical cyclones in the Caribbean basin -Rodent-borne infections after flooding and droughts	Limited adaptive capacity due to high infant mortality, income inequalities, weak health care system.
Small Island developing states	-Thermal stresses due to heat waves -Transmission of vector-borne diseases to new areas and increased morbidity and mortality due to diarrhea -Increased deaths and injuries due to tropical cyclones	Low adaptive capacity, due to poor resources, weak health care system and high frequency of natural hazards
Europe	-Thermal stresses due to heat waves -Air pollution related diseases -Increased deaths and injuries due to extreme events and flooding -Expected increase in lyme disease and tick-borne encephalitis in temperate regions -Expected increase in leishmaniasis in Mediterranean countries	Adaptive capacity higher than in developing countries. Existing public health resources will allow to put in place curative and preventive measures to face at least part of the health impacts.
North America	-Thermal stresses due to heat waves, mainly in Nord-east and Mid-west -Injuries and mortality due to storms, floods, hurricanes, tornadoes and ice storms -Increased vector and water-borne diseases	Adaptive capacity higher than in developing countries. Existing public health resources will allow to put in place curative and preventive measures to face at least part of the health impacts.
Australia and New Zealand	-Thermal stresses due to heat waves -Air pollution related diseases -Increased deaths and injuries due to tropical cyclones and floods -Increased transmission of vector-borne diseases (dengue)	Adaptive capacity higher than in developing countries. Existing public health resources will allow to put in place curative and preventive measures to face at least part of the health impacts.

Source: adapted from UNFCCC 2007 [10].

**Table 3.** Health Adaptation Measures to Climate Change.

Adaptation measures		Health impacts				
		Thermal stresses	Extreme weather events	Vector-borne diseases	Water-borne diseases	Food-borne diseases
Legislative and regulatory	Anticipatory	- Building guidelines	- Building guidelines - Economic incentives for building - Urban planning regulation - Forced migration		- Watershed protection laws - Water quality and water supply regulation	- Food sanitation and hygiene regulation
Technical	Anticipatory	- Urban planning (green islands, fountains, greenroofs) - Thermal building insulation and air conditioning	- Urban planning (flood-resistant) - Flood protection elevation - Flood protection structures (dams, dykes, walls and raised banks, pump stations) - Reforestation	- Vector control - Vaccination, impregnated bed nets - Surveillance, prevention and control programs - Epidemic forecasting	- Water treatment and distribution - Monitoring water sources - Regulated piped water in houses - Improved sanitation (latrines) - Household sewer connection - Surveillance, prevention and control programs	- Refrigeration - Chlorination of drinking water - Pasteurization of milk - sanitary slaughter and processing o meat, poultry and seafood
	Reactive	- Financial and domiciliary assistance services, “telecare” systems, accompaniment and transport to emergency medical services - Emergency plans (hospital and primary care)	- Pre-disaster recovery plans - First aid and emergency plans - Temporary evacuation	- Hospital and primary care - Outreach doctors	- Hospital and primary care - Outreach doctors	- Food-borne disease surveillance - Hospital and primary care - Outreach doctors
Education and advisory	Anticipatory	- Heat watch warning systems - Educational campaign	- Real-time forecasting - Early warning systems - Educational campaign	- Education campaign	- Health educational campaigns - Boil water alerts	- Food safety education
Cultural and behaviour	Anticipatory	Clothing, drinking, visiting places with air conditioning and green areas	- Use of storm shelters	- Water storage practices	- Washing hands and hygiene - Use of pit latrines	- Avoid high risky food (such as runny eggs and raw shellfish) - Separating cooked and raw food - Wash hands, cutting boards and contaminated surfaces

Sources: adapted from [12] and [15].

**Table 4.** Annual Costs of Health Adaptation to Climate Change. Worldwide 2000–2030 (US$Million, 2000).

Cost/Scenario	Unmitigated	Stabilization at 750ppm	Stabilization at 550ppm
Malaria	3,100 to 8,800	1,900 to 5,600	1,600 to 4,500
Diarrhea	2,731 to 9,010	1,983 to 6,814	1,706 to 6,024
Malnutrition	62 to 166	81 to 216	54 to 150
All Costs	5,900 to 18,000	4,000 to 12,600	3,300 to 10,700

**Table 5.** Additional Annual Costs of Health Adaptation in Alternative Climate Change Scenarios per Geographical World Region 2000–2030 (Million US$, 2000).

REGION	Climate Scenario					
	S550	S750	UE	S550	S750	UE
	Diarrhea			Malaria		
*Developing countries*						
Africa	633–1,334	756–1,646	954–2,026	1,283–3,718	1,567–4,595	2,508–7,222
Americas (Central/South)	22–372	22–442	22–582	23–65	29–76	43–121
Eastern Mediterranean	87–713	87–765	131–1,122	230–626	284–772	434–1,231
South East Asia	952–2,198	1,106–2,542	1,428–3,231	0–8	6–9	6–17
Western Pacific (A)	0–1,109	0–1,109	185–1,664	37–98	43–120	68–188
*Subtotal*	*1,694–5,726*	*1,971–6,504*	*2,719–8,625*	*1,573–4,514*	*1,928–5,572*	*3,059–8,780*
*Developed countries*						
North America	0–70	0–70	0–94	0	0	0
Europe	12–205	12–217	12–260	0	0	0
Western Pacific (B)	0–23	0–23	0–32	0.136–0.370	0.177–0.494	0.265–0.741
*Subtotal*	*12–298*	*12–310*	*12–385*	*0.253*	*0.335*	*0.503*
WORLD	*1,706–6,024*	*1,983–6,814*	*2,731–9,010*	*1,573–4,515*	*1,928–5,573*	*3,059–8,781*

Source: based on cost estimates from Ebi [19]. Note: S550 implies stabilization of emissions of GHGs at 550 ppm by 2210. S750 implies stabilization of emissions at 750 ppm by 2170 and UE implies unmitigated emissions. Note: See Annex 1 reporting the member states in each region.

**Table 6.** Annual Costs of Health Interventions to Reduce Malaria 2006–2015 (Million US$, 2006).

REGION	Annual Costs
Africa	1,707–2,186
Americas (Central/South)	212–235
South East Asia, Western Pacific and Eastern Mediterranean	1,903–4,638
Total	*3,823–4,638*

Source: based on cost estimates from Kiszewski *et al.* [20].

They refer to a 50% reduction by 2010 and a 75% reduction by 2015.

**Table 7.** Annual Costs for Water and Sanitation Programs 2000–2015 and Expected Cases Avoided (Million US$, 2000).

REGION	Annual Costs by Intervention				
	Halving proportion people without access to improved water	Halving proportion of people without access to improved water and sanitation	Access for all to improved water and sanitation	Access for all to improved water and sanitation, with water disinfected	Access for all to regulated piped water and sewage connection at home
Developing countries					
Africa	490	2,021	4,043	4,360	24,729
Americas					
(Central/South)	171	788	1,577	1,937	14,085
Eastern					
Mediterranean	57	263	526	633	7,329
South East Asia	403	4,094	8,190	8,762	47,238
Western Pacific (A)	565	3,621	7,243	7,686	32,767
*Subtotal*	*1,686*	*10,787*	*21,579*	*23,378*	*126,148*
*Percent of cases avoided*	*3%*	*10%*	*17%*	*54%*	*70%*
Developed countries					
North America	0	0	1	1	2
Europe	77	369	738	965	9,464
Western Pacific (B)	19	147	294	304	900
*Subtotal*	*96*	*516*	*1,033*	*1,270*	*10,366*
*Percent of cases avoided*	*1%*	*3%*	*6%*	*36%*	*49%*
WORLD	*1,782*	*11,303*	*22,612*	*24,648*	*136,514*

Source: based on cost estimates from Hutton and Haller [27].

**Table 8.** Studies about the Costs of Health Intervention Programs Relevant to Climate Change.

Study	Coverage	Annual costs of health interventions in developing countries (Million US$)	Cost per Case or Per Death Avoided (US$)	Comments/Intervention
Costs of health adaptation to climate change				
Ebi (2008) [19]	Malaria, diarrhea, malnutrition.	(US$ 2000) 3,100–8,800 (malaria) 2,700–9,000 (diarrhea) 62–166 (malnutrition)	-	Intervention program from 2000 to 2030. Prevention and treatment measures. Different scenarios for climate investigated. Worldwide.
Van Rensburg and Blignaut (2002) [21]	Malaria.	3,800 (US$ 2000)	-	Intervention program from 2000 to 2025. Prevention and treatment measures to achieve 95% coverage. Different malaria risk scenarios. South Africa.
Costs of climate-related disease control programs in the public health context				
Kiszewski et al (2007) [20]	Malaria	3,823–4,638 (US$ 2006)	257–296 per case avoided (US$ 2006) Estimate is based on S. America data only.	Intervention program 2006–2015 to achieve 80% coverage and 75% reduction in cases by 2015. Treatment/prevention and support activities programs. Africa, Asia and Middle East, South America.
Epstein and Mills (2005) [23]	Malaria	(US$ 2005) 70% coverage by 2015: 1,400–2,500 (treatment) 1,450–3,200 (prevention) 40%–50% coverage by 2010: 600–1,100 (treatment) 650–1,400 (prevention)	-	Interventions in Sub Saharan Africa to achieve the Millennium Development Goals (MDGs): 40% coverage for prevention and 50% for treatment by 2010. 70% coverage for treatment and prevention by 2015.
Morel et al (2005) [25]	Malaria	468 (US$ 2000) (Western Africa) 442 (US$ 2000) (Southern and Eastern Africa)	-	Intervention program for 10 years. Combined therapy of preventive and treatment measures. Sub-Saharan Africa.
Stenberg *et al.* (2007) [26]	Malnutrition, diarrhea, malaria, pneumonia and newborn diseases.	2,200–7,800 (US$ 2006)	314–630 per death avoided (US$ 2006)	Intervention program 2006–2015 to reduce child mortality and morbidity by 2/3 by 2015 (MDGs). Prevention and treatment. All costs included. 75 developing countries.
Hutton and Haler (2004) [27]	Diarrhea (cholera, salmonellosis, shigellosis, other intestinal infections).	1,782–136,514 (US$ 2000)	11.5–36.7 per case avoided (US$ 2000).	Structural intervention program for water and sanitation 2000–2015. Different scenarios of increased access. Worldwide.
Meddings et al (2004) [28]	Diarrhea	0.5–1 (US$ 1999)	1,804–4,086 per child death avoided (US$ 1999)	Structural interventions: latrine construction and rehabilitation program in Kabul (Afganistan).

**Table 9.** Annual Cost per Case of Diarrhea Avoided with Water and Sanitation Programs 2000–2015 (US$, 2000).

Annual Cost per Case Avoided by Intervention Scenario (US$, 2000)				
Halving proportion people without access to improved water	Halving proportion of people without access to improved water and sanitation	Access for all to improved water and sanitation	Access for all to improved water and sanitation, with water disinfected	Access for all to regulated piped water and sewage connection at home
11.52	20.71	25.04	8.61	36.72

Source: based on cost estimates from Hutton and Haller [27].

**Table 10.** Studies about the Cost-Effectiveness of Health intervention Programs in Developing Countries.

Study	Coverage	Cost per Death (DA) or Case Avoided (CA) (US$)	Comments/Intervention
Martines *et al.* (1993) [31]	Diarrhea	226 (US$ 1999) (DA)	Immunization program. Indonesia and Ghana
Shepard (1986) [32]	Diarrhea	887 (US$ 1999) (DA)	Immunization program. Côte d’Ivoire.
USAID Micronutrient program (2004) [33]	Diarrhea	236 (US$ 1999) (DA)	Standard child health intervention. Vitamin A supplementation. Ghana, Nepal, Zambia.
Horton (1996) [34]	Diarrhea	(US$ 1999) (DA) 115–625 (Brazil) 919 (Honduras) 174–216 (Mexico)	Breastfeeding promotion. Brazil, Honduras, Mexico.
Martines *et al.* (1993) [31]	Cholera	(US$ 1999) 273 (CA)	Routine cholera immunization. Bangladesh.
Graves *et al.* (1998) [35]	Malaria	(US$ 1999) (DA) 305 (vaccine) 858 (net impregnation)	Malaria prevention: vaccination and insecticide impregnation of bed nets. Gambia.
Picard *et al*. (1993) [36]	Malaria	(US$ 1999) (DA) 227–410 (net impregnation) 683 (net impregnation/chemoprophylaxis)	Malaria prevention: insecticide impregnation of bed nets and chemoprophylaxis. Gambia.
Aikins *et al*. (1998) [37]	Malaria	537 (US$ 1999) (DA)	Malaria prevention: bed net impregnation. Gambia.
Loevinsohn (1997) [38]	Malaria	73–279 (US$ 1999) (DA)	Malaria prevention: vitamin A supplementation. Philippine.
Fiedler (2000) [39]	Malaria	302–414 (US$ 199) (DA)	Malaria prevention: vitamin A supplementation. Nepal.
Schulz *et al*. (1995) [40]	Malaria	81–950 (US$ 1995) (DA)	Malaria prevention in pregnancy: antenatal treatment and chemoprophylaxis. Malawi.
Akhavan *et al*. (1999) [41]	Malaria	271–1,355 (US$ 1995) (DA)	Malaria treatment: early diagnosis and prompt treatment. Brazil.

## Appendix

**Annex 1.** WHO Regions.

Region	Description	Member States
Africa	Developing countries with high mortality	Algeria, Angola, Benin, Burkina Faso, Cameroon, Cape Verde, Chad, Comoros, Equatorial Guinea, Gabon, Gambia, Ghana, Guinea, Guinea-Bissau, Liberia, Madagascar, Mali, Mauritania, Mauritius, Niger, Nigeria, San Tome and Principe, Senegal, Seychelles, Sierra Leone, Togo Botswana, Burundi, Central African Republic, Congo, Cote d’Ivoire, Democratic Republic of the Congo, Eritrea, Ethiopia, Kenya, Lesotho, Malawi, Mozambique, Namibia, Rwanda, South Africa, Swaziland, Uganda, United Republic of Tanzania, Zambia, Zimbabwe
North America	Developed countries with very low mortality	Canada, Cuba, United States of America
Central and South America	Developing countries with low mortality Developing countries with high mortality	Antigua and Barbuda, Argentina, Bahamas, Barbados, Belize, Brazil, Chile, Colombia, Costa Rica, Dominica, Dominican Republic, El Salvador, Grenada, Guyana, Honduras, Jamaica, Mexico, Panama, Paraguay, Saint Kitts and Nevis, Saint Lucia, Saint Vincent and the Grenadines, Suriname, Trinidad and Tobago, Uruguay, Venezuela (Bolivarian Republic of) Bolivia, Ecuador, Guatemala, Haiti, Nicaragua, Peru
South East Asia	Developing countries with low mortality Developing countries with high mortality	Indonesia, Sri Lanka, Thailand Bangladesh, Bhutan, Democratic People s Republic of Korea, India, Maldives, Myanmar, Nepal, Timor-Leste
Europe	Developed countries with very low mortality Developed countries with low mortality Developed countries with low child and high adult mortality	Andorra, Austria, Belgium, Croatia, Cyprus, Czech Republic, Denmark, Finland, France, Germany, Greece, Iceland, Ireland, Israel, Italy, Luxembourg, Malta, Monaco, Netherlands, Norway, Portugal, San Marino, Slovenia, Spain, Sweden, Switzerland, United Kingdom Albania, Armenia, Azerbaijan, Bosnia and Herzegovina, Bulgaria, Georgia, Kyrgyzstan, Poland, Romania, Serbia and Montenegro, Slovakia, Tajikistan, The former Yugoslav Republic of Macedonia, Turkey, Turkmenistan, Uzbekistan Belarus, Estonia, Hungary, Kazakhstan, Latvia, Lithuania, Republic of Moldova, Russian Federation, Ukraine
Eastern Mediterranean	Developing countries with low mortality Developing countries with high mortality	Bahrain, Iran (Islamic Republic of), Jordan, Kuwait, Lebanon, Libyan Arab Jamahiriya, Oman, Qatar, Saudi Arabia, Syrian Arab Republic, Tunisia, United Arab Emirates Afghanistan, Djibouti, Egypt, Iraq, Morocco, Pakistan, Somalia, Sudan, Yemen
Western Pacific (A)	Developed countries with very low mortality	Australia, Brunei Darussalam, Japan, New Zealand, Singapore
Western Pacific (B)	Developing countries with low mortality	Cambodia, China, Cook Islands, Fiji, Kiribati, Lao People s Democratic Republic, Malaysia, Marshall Islands, Micronesia (Federated States of), Mongolia, Nauru, Niue, Palau, Papua New Guinea, Philippines, Republic of Korea, Samoa, Solomon Islands, Tonga, Tuvalu, Vanuatu, Viet Nam

Source: adapted from WHO 2002 [42].

## References

[1] IPCC Climate Change 2007: Impacts, Adaptation and VulnerabilityWorking Group II Contribution to the IPCC, 2007. Available at http://www.gtp89.dial.pipex.com/chpt.htm(accessed December 29, 2008)

[2] McMichael AJ, Campbell-Lendrum D, Kovats S, Edwards S, Wilkinson P, Wilson T, Nicholls R, Hales S, Tanser F, LeSueur D, Schlesinger M, Andronova N, Ezzati M, Lopez A, Rodgers A, Murray C (2004). Global Climate Change. Comparative Quantification of Health Risks: Global and Regional Burden of Disease due to Selected Major Risk Factors.

[3] Epstein Y, Sohar E (1995). Shapiro, Y. Exceptional Heatstroke: a preventable condition. Israel J. Med. Sci.

[4] Confalonieri U Menne B Akhtar R Ebi KL Hauengue M Kovats RS Revich B Woodward A Human Health 2007 Climate Change 2007: Impacts, Adaptation and VulnerabilityContribution of Working Group II to the Fourth Assessment Report of the Intergovernmental Panel on Climate Chang 2007 Available at http://www.gtp89.dial.pipex.com/chpt.htm(accessed December 29, 2008)

[5] Langford IH, Bentham G (1995). The potential effects of climate change on winter mortality in England and Wales. Int. J. Biometeorol.

[6] Martens WJM (1997). Climate change, thermal stress and mortality changes. Soc. Sci. Med.

[7] Armstrong B, Mangtani P, Fletcher A, Kovats RS, McMichael AJ, Pattenden S, Wilkinson P (2004). Effect of influenza vaccination on excess deaths occurring during periods of high circulation of influenza: cohort study in elderly people. Brit. Med. J.

[8] Christensen JH, Hewitson B, Busuioc A, Chen A, Gao X, Held I, Jones R, Kolli RK, Kwon WT, Laprise R, Magaña Rueda V, Mearns L, Menéndez CG, Räisänen J, Rinke A, Sarr A, Whetton P (2007). Regional Climate Projections. Climate Change 2007: The Physical Science Basis; Contribution of Working Group I to the Fourth Assessment Report of the Intergovernmental Panel on Climate Change.

[9] Martens WJM, Jetten TH, Rotmans J, Niessen LW (1995). Climate change and vector-borne diseases: a global modelling perspective. Global Environ. Change.

[10] UNFCCC (2007). Climate Change: Impacts, Vulnerabilities and Adaptation in Developing Countries.

[11] European Environment Agency EEA Costs of Adaptation to Climate Change: A Review of Assessment Studies With a Focus on Methodologies UsedAgreement No 3602/B2005, EEA under the Framework Contract No EEA/AIR/04/004, Final working paper2007

[12] IPCC TAR Climate Change 2001: Impacts, Adaptation and VulnerabilityIPCC Third Assessment ReportCambridge University Press New York, NY, USA 2001

[13] McMichael AJ, Kovats RS (2000). Climate change and climate variability: adaptations to reduce adverse health impacts. Environ. Monit. Assess.

[14] European Environment Agency EEA Climate Change: the Cost of Inaction and the Cost of Adaptation2007, EEA Technical Report No. 13/2007. ISSN 1725–2237EEA Copenhagen K, Denmark 2007

[15] Alberini A Chiabai A Valutazione degli Impatti dei Cambiamenti Climatici sulla SaluteAgenzia per la Protezione dell’Ambiente e per i Servizi Tecnici APAT and Centro Euro Mediterraneo per i Cambiamenti Climatici CMCC2007

[16] Ortiz R Cost per Quality-adjusted Life Year and Thresholds in the UK, Ireland, Norway, Sweden and FinlandPaper Prepared for the EU NEEDS Project, 2005; Available at http://www.needsproject.org/index.php?option=com_content&task=view&id=42&Itemid=66(accessed December 28, 2008)

[17] Bosello F, Roberto R, Tol RSJ (2006). Economy-wide Estimates of the Implications of Climate Change: Human Health. Ecol. Econ.

[18] Stern N Stern Review on the Economics of Climate Change Cambridge University Press The Edinburgh Building, Cambridge CB2 8RU, UK 2006Available at http://www.hmtreasury.gov.uk/independent_reviews/stern_review_economics_climate_change/sternreview_report.cfm(accessed December 28, 2008)

[19] Ebi KL (2008). Adaptation Costs for Climate Change-Related Cases of Diarrhoeal Disease, Malnutrition, and Malaria in 2030. Global Health.

[20] Kiszewski A, Johns B, Schapria A, Delacollette C, Crowell V, Tan-Torres T, Ameneshewa B, Teklehaimanot A, Nafo-Traore F (2007). Estimated Global Resources Needed to Attain International Malaria Control Goals. Bulletin WHO.

[21] Van Rensburg JJJ, Blignaut JN The Economic Impact of an Increasing Health Risk due to Global Warming.

[22] Mills A, Targett GAT (1991). The economics of Malaria control. Waiting for the vaccine.

[23] Epstein Y, Mills E (2005). Climate Change Futures. Health, Ecological and Economic Dimensions.

[24] Murray C, Lopez A (1996). Global Burden of Disease.

[25] Morel CM, Lauer JA, Evans DB (2005). Cost Effectiveness Analysis of Strategies to Combat Malaria in Developing Countries. BMJ Bri. Med. J.

[26] Stenberg J, Johns B, Scherpbier RW, Edeger TT-T (2007). A Financial Road Map to Scaling Up Essential Child Health Interventions in 75 Countries. Bulletin WHO.

[27] Hutton G Haller L Evaluation of the Costs and Benefits of Water and Sanitation Improvements at the Global Level World Health Organization Geneva, Switzerland 2004WHO/SDE/WSH/04.04

[28] Meddings DR, Ronald LA, Marion S, Pinera JF, Oppliger A (2004). Cost Effectiveness of a latrine revision programme in Kabul, Afghanistan. Bulletin WHO.

[29] Goodman CA Coleman P Mills AJ Economic Analysis of Malaria Control in Sub-Saharan Africa Global Forum for Health Research Geneva, Switzerland 2000ISBN 2-940286-00-0

[30] Goodman CA, Mills AJ (1999). The Evidence Base on the Cost-Effectivenss of Malaria Control Measures in Africa. Health Policy Plan.

[31] Martines J, Phillips M, Feachem RG, Jamison DT, Measham AR, Mosley WH, Bodadilla JL (1993). Diarrheal Diseases. Disease Control Priorities in Developing Countries.

[32] Shepard DS, Sanoh L, Coffi E (1986). Cost-Effectiveness of the Expanded Program on Immunization in the Ivory Coast: A Preliminary Assessment. Soc. Sci. Med.

[33] USAID (United States Agency for International Development) Micronutrient Program (2004). Cost analysis of the national vitamin A supplementation programs in Ghana, Nepal, and Zambia: A synthesis of three studies.

[34] Horton S, Sanghvi T, Phillips M, Fiedler J, Perez-Escamilla R, Lutter C, Rivera A, Segall-Correa AM (1996). Breastfeeding Promotion and Priority Setting in Health. Health Policy Plan.

[35] Graves PM (1998). Comparison of the Cost-Effectiveness of Vaccines and Insecticide Impregnation of Mosquito Nets for the Prevention of Malaria. Ann. Trop. Med. Parasitol.

[36] Picard J, Aikins M, Alonso PL (1993). A malaria Control Trial Using Insecticide-Treated Chemoprophylaxis in a Rural Area of The Gambia, West Africa. Cost-effectiveness of Bed Net Impregnation Alone or Combined With Chemopropylaxis in Preventing Mortality and Morbidity from Malaria in Gambian Children. Trans. R. Soc. Trop. Med. Hyg.

[37] Aikins MK, Fox-Rushby J, D’Alessandro U, Langerock P, Cham K, New L, Bennett S, Greenwood B, Mills A (1998). The Gambian National Impregnated Bednet Programme: Consequences and Net Cost-Effectiveness. Soc. Sci. Med.

[38] Loevinsohn BP, Sutter RW, Costales MO (1997). Using Cost-Effectiveness Analysis to Evaluate Targeting Strategies: the Case of Vitamin A Supplementation. Health Policy Plan.

[39] Fiedler JL (2000). The Nepal National Vitamin A Program: Prototype to Emulate or Donor Enclave?. Health Policy Plan.

[40] Schultz LJ, Steketee RW, Chitsulo L, Wirima JJ (1995). Antimalarials during Pregnancy: a Cost- Effectiveness Analysis. Bulletin WHO.

[41] Akhavan D, Musgrove P, Abrantes A, d’A Gusmão R (1999). Cost-effective Malaria Control in Brazil: Cost-effectiveness of a Malaria Control Program in the Amazon Basin of Brazil, 1988–1996. Soc. Sci. Med.

[42] World Health Organization (WHO) (2002). The world health report 2002.

